# When 3 Fundamentals of Disaster Meet During Elective PCI

**DOI:** 10.1016/j.jaccas.2024.103126

**Published:** 2025-02-05

**Authors:** Max Wagener, Jasper Boeddinghaus, Gregor Leibundgut

**Affiliations:** University Heart Center Basel, University Hospital Basel, Basel, Switzerland

**Keywords:** balloon tamponade, coronary coiling, coronary perforation, PCI, snaring device, stent dislodgement, stent graft, vessel rupture

## Abstract

The disaster risk is probabilistically defined as a function of hazard, vulnerability, and capacity. Here, we report a case of a vulnerable patient who underwent elective percutaneous coronary intervention for symptomatic ischemic heart disease in whom a series of 5 percutaneous coronary intervention–related hazards occurred: distal coronary perforation; ischemia-driven electrical storm; no reflow; coronary vessel rupture; and stent dislodgement. These complications were managed using several interventional techniques including immediate balloon occlusion, distal vessel coiling, drug-eluting stent implantation, recovery of a dislodged drug-eluting stent on the fractured wire using a self-made snare and finally crushing of a stent graft against the vessel wall. Despite these efforts, prolonged resuscitation and limited patient-related capacities were unfavorable and the patient died due to cardiogenic shock.

## Visual Summary

**Figure:**
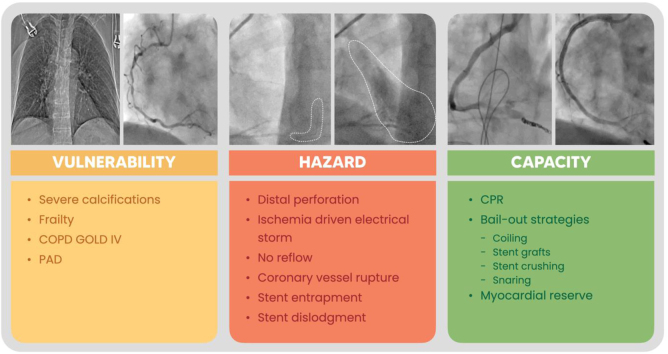

The World Health Organization and the United Nations define that the risk of disaster is probabilistically defined as a function of hazard, vulnerability, and capacity.^1^^,^^2^ Here, we present a case of a patient with ischemic heart disease undergoing elective percutaneous coronary intervention (PCI), where all 3 conditions were met and eventually led to a fatal outcome.

**Take-Home Messages**

• Screen PCI candidates for patient-specific and lesion-specific vulnerability and balance the interventional risk against the estimated health gain.
• Know your interventional bailout strategies and material available in your lab.
• Share and discuss your complications with peers in to improve awareness and reduce the risk of disastrous outcomes.

## Vulnerability

**Figure 1:**
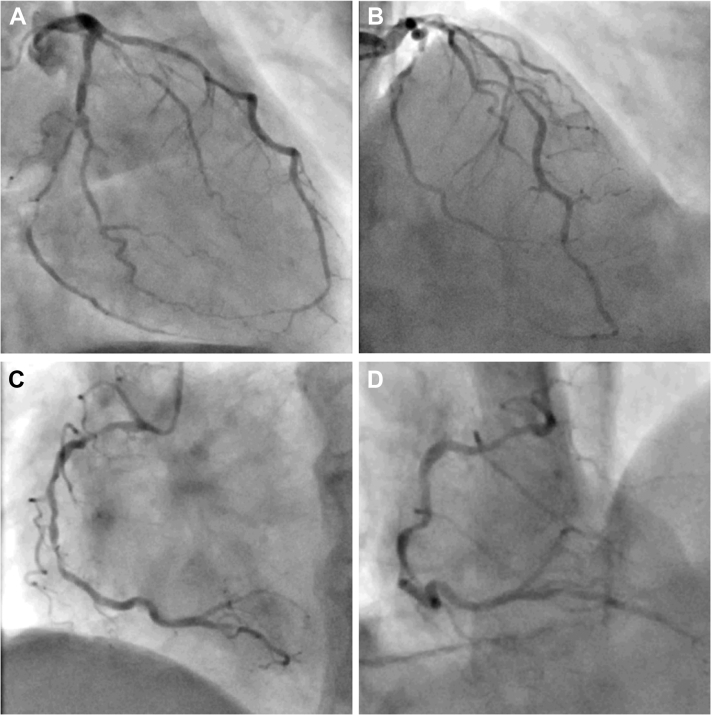
Angiographic Lesion Assessment (A) Left coronary system with serial stenoses of the right circumflex artery. (B) Left coronary system with stenosis of the mid left anterior descending artery. (C) Right coronary artery with serial severe stenoses of the mid segment. (D) Tortuosity of the right coronary artery.

A 74-year-old female patient with a moderate (19%) European Society of Cardiology risk factor–weighted clinical likelihood of obstructive coronary artery disease was referred for elective PCI after proof of inferior ischemia and transitory ischemic dilatation during myocardial perfusion scintigraphy.^3^ Besides classical cardiovascular risk factors (type 2 diabetes, hypertension, smoking), her past medical history was significant for severe peripheral artery disease with stenting of the right external iliac artery, chronic obstructive cardiopulmonary disease GOLD (Global Initiative for Chronic Obstructive Lung Disease) stage IV, osteoporosis, and unstable gait of multifactorial origin. Coronary angiography via the right radial access showed significant coronary artery disease with high-grade stenosis of the left circumflex artery (Figures 1A and 1B) and serial high-grade stenoses of the right coronary artery (RCA) (Figures 1C and 1D).

## PCI

Wiring of the RCA was not possible with a workhorse wire (Sion Blue, ASAHI Intecc Co Ltd) and after successful lesion crossing with a polymer-jacketed guidewire (Sion Black), predilatation using a 2.5 semi-compliant (SC) balloon (Artimes, Brosmed) was performed.

## Hazard 1

**Figure 2:**
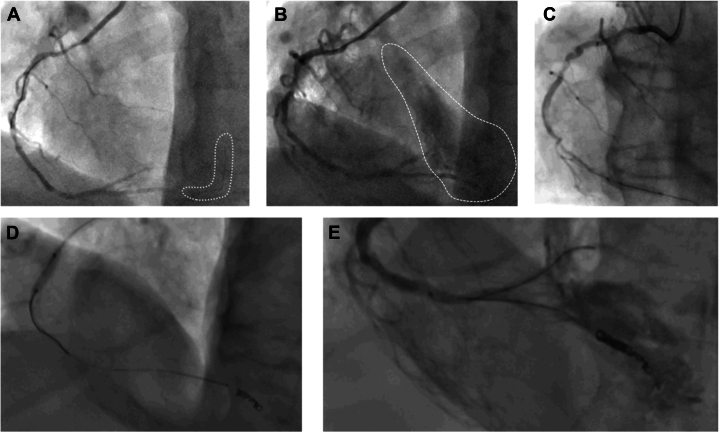
Management of Distal Wire Perforation (A) Distal perforation of Sion Black wire. (B) Distal vessel perforation and bleeding. (C) Balloon occlusion of the posterior descending artery. (D) Coiling of the distal posterior descending artery. (E) Perforation of the posterior descending artery with intramyocardial bleeding.

After difficult predilatation of the severely calcified RCA, a distal wire perforation of the posterior descending artery (PDA) was noted (Figures 2A and 2B). Balloon occlusion of the PDA with the 2.5 SC balloon (Figure 2C) was unsuccessful. Due to ongoing bleeding from the perforation, predilatation of the RCA with the 2.5 SC balloon was performed to allow parallel wiring with a workhorse wire (Sion Blue) and placing of a total of 4 Azur CX coils (Terumo Corporation) through a Progreat microcatheter (Terumo Corporation) (Figure 2D).

## Hazard 2

Following predilatation of the mid RCA, a flow-limiting dissection occurred. The combination of the dissection and the prolonged balloon occlusion of the PDA resulted in an ischemia-driven electrical storm. Consequently, mechanical cardiopulmonary resuscitation was initiated, and more than 10 consecutive defibrillations were performed. At the same time, flow in the coronary artery could be restored by implanting 2 drug-eluting stents (DES) (3.0 × 48 mm and 3.5 × 12 mm; Synergy XD, Boston Scientific) in the proximal to mid-RCA.

## Hazard 3

**Figure 3:**
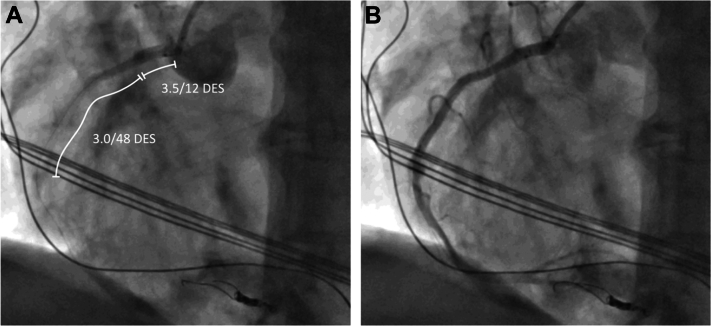
Acute Stent Thrombosis (A) Acute stent thrombosis, with no reflow. (B) Reperfusion after administration of additional heparin. DES = drug-eluting stent.

After successful implantation of the DES, acute stent thrombosis occurred and resulted in another run of ventricular fibrillation that had to be defibrillated. The stent thrombosis could be reverted by giving an additional 5,000 U of unfractionated heparin (Figures 3A and 3B).

## Hazard 4

**Figure 4:**
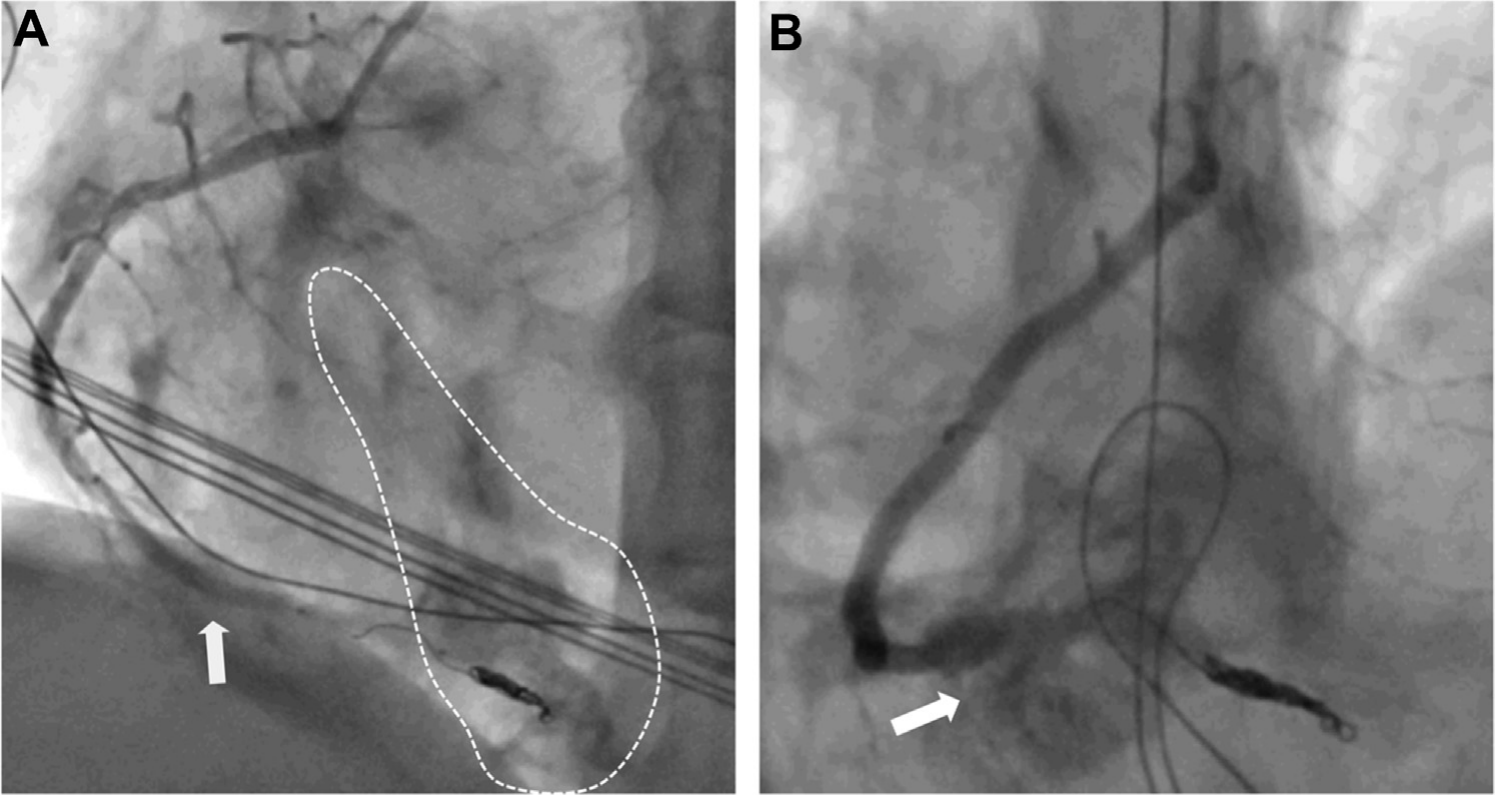
Vessel Rupture (A) Ongoing bleeding from the distal perforation (dashed line) despite 3.0 balloon (white arrow). (B) Vessel rupture (white arrow) due to upsizing to 3.5 balloon.

Although there were no signs of pericardial tamponade and bedside echocardiography showed only minor effusion, ongoing bleeding into the myocardium and epicardial fat tissue from the distal wire perforation in the PDA was noted on the angiogram (Figure 2E). Additional balloon occlusion with a larger 3.5 SC balloon was performed, which was complicated by vessel rupture at the crux (Figures 4A and 4B).

## Hazard 5

**Figure 5:**
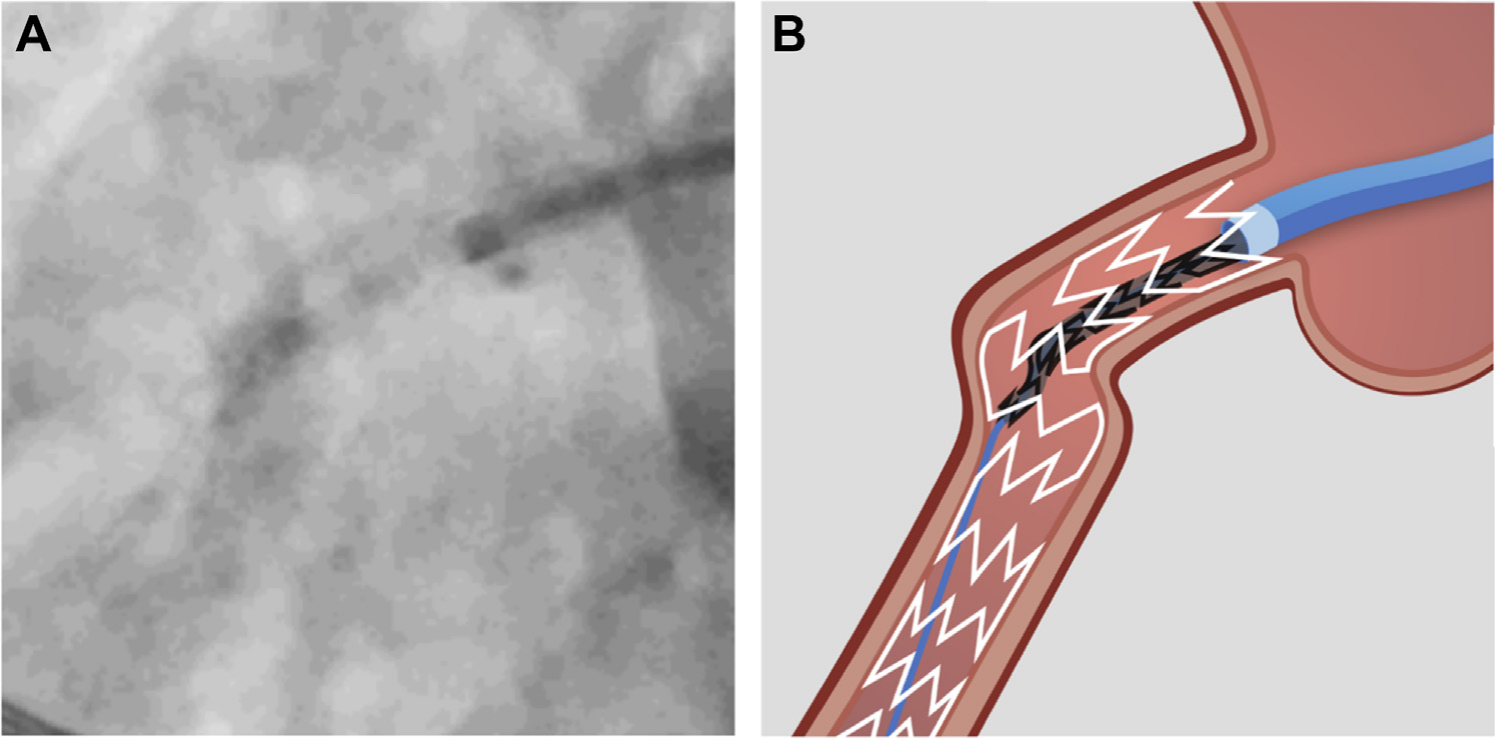
Dislodged Stent Graft (A) Angiogram of the dislodged stent graft. (B) Illustration of the dislodged stent graft. White = DES in place; black = dislodged stent graft; blue = Amplatz Left 0.75 guide catheter. Abbreviation as in Figure 3.

**Figure 6:**
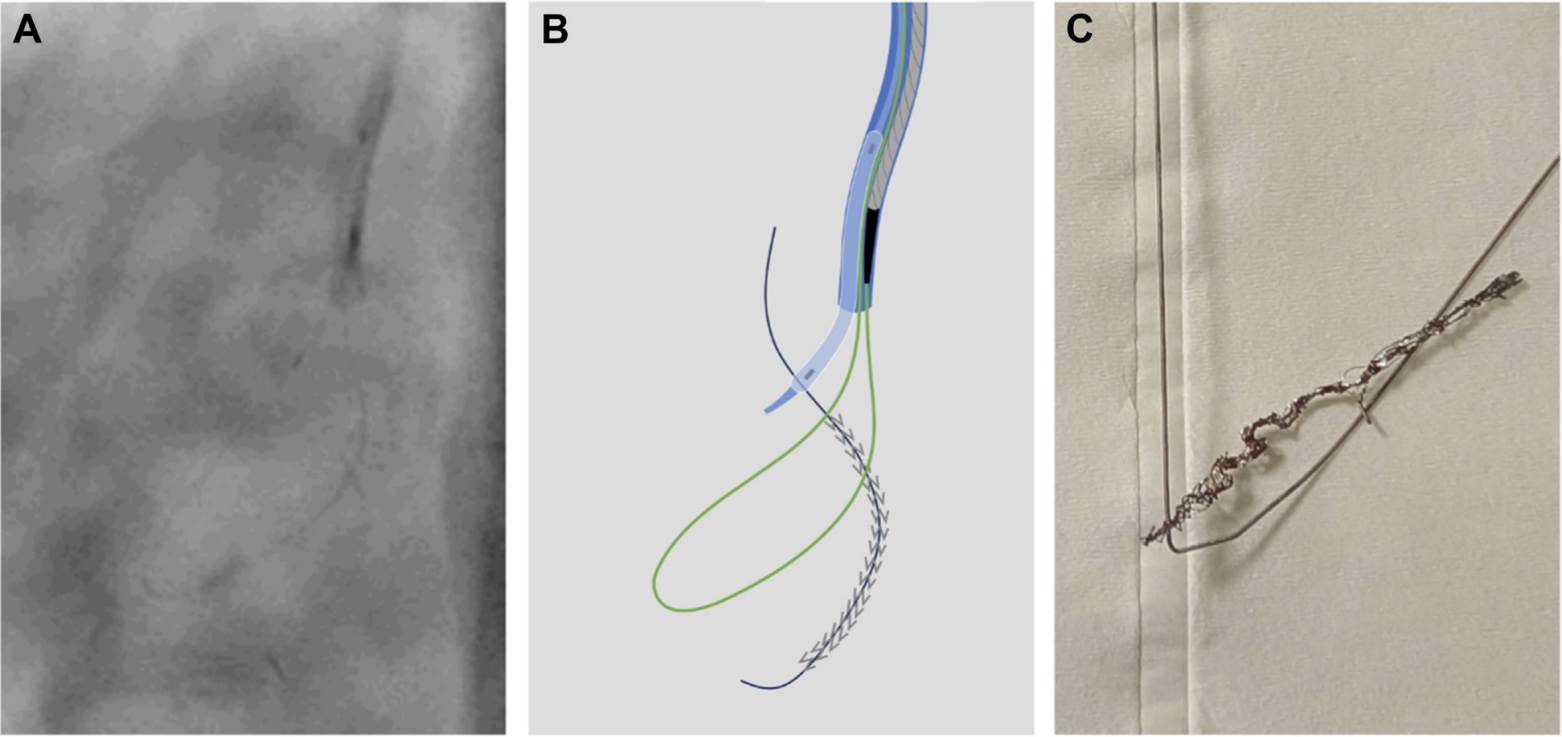
Self-Made Snare (A) Angiogram of the snare used to recover the dislodged DES. (B) Illustration of snaring the stent (gray) on the guidewire (black) using a 6-F Amplatz Left 0.75 guide catheter (blue) and a Sion Blue wire (green) through a microcatheter (gray) pinned inside the guide catheter using a 2.5 semi-compliant balloon (blue). (C) Picture of the recovered stent and fractured Sion Blue wire. Abbreviation as in Figure 3.

An attempt to cover the vessel rupture by deploying a covered 3.0 × 24–mm stent graft (BeGraft, Bentley Endovascular Group AB) was unsuccessful. The graft got trapped within the DES in the proximal RCA and dislodged from the balloon (Figures 5A and 5B). Small balloon technique with a 1.0 SC balloon was not possible, thus the decision to crush the graft to the vessel wall with another DES was made.^4^ Rewiring with a Sion Blue was successful, but the delivery of a 3.0 × 28-mm DES (Synergy XD) failed. Eventually, the DES was also lost next to the stent graft, and the Sion Blue got trapped and fractured. Alternative arterial access to snare and recover the stent on the fractured wire was not possible due to extensive peripheral artery disease, previous femoro-iliac stents, and high-grade bifemoral stenosis with protruding calcific plaques. Thus, the primary right radial access was used to recover the DES. The guide catheter was removed from the patient, flushed, and reinserted over the same access to prevent embolization of unraveled coils potentially remaining within the guide catheter. With no dedicated large loop snare at hand, a self-made snare was used to recover the distorted, elongated stent and the remaining part of the fractured Sion Blue (Figures 6A to 6C).^5^

## Capacity

**Figure 7:**
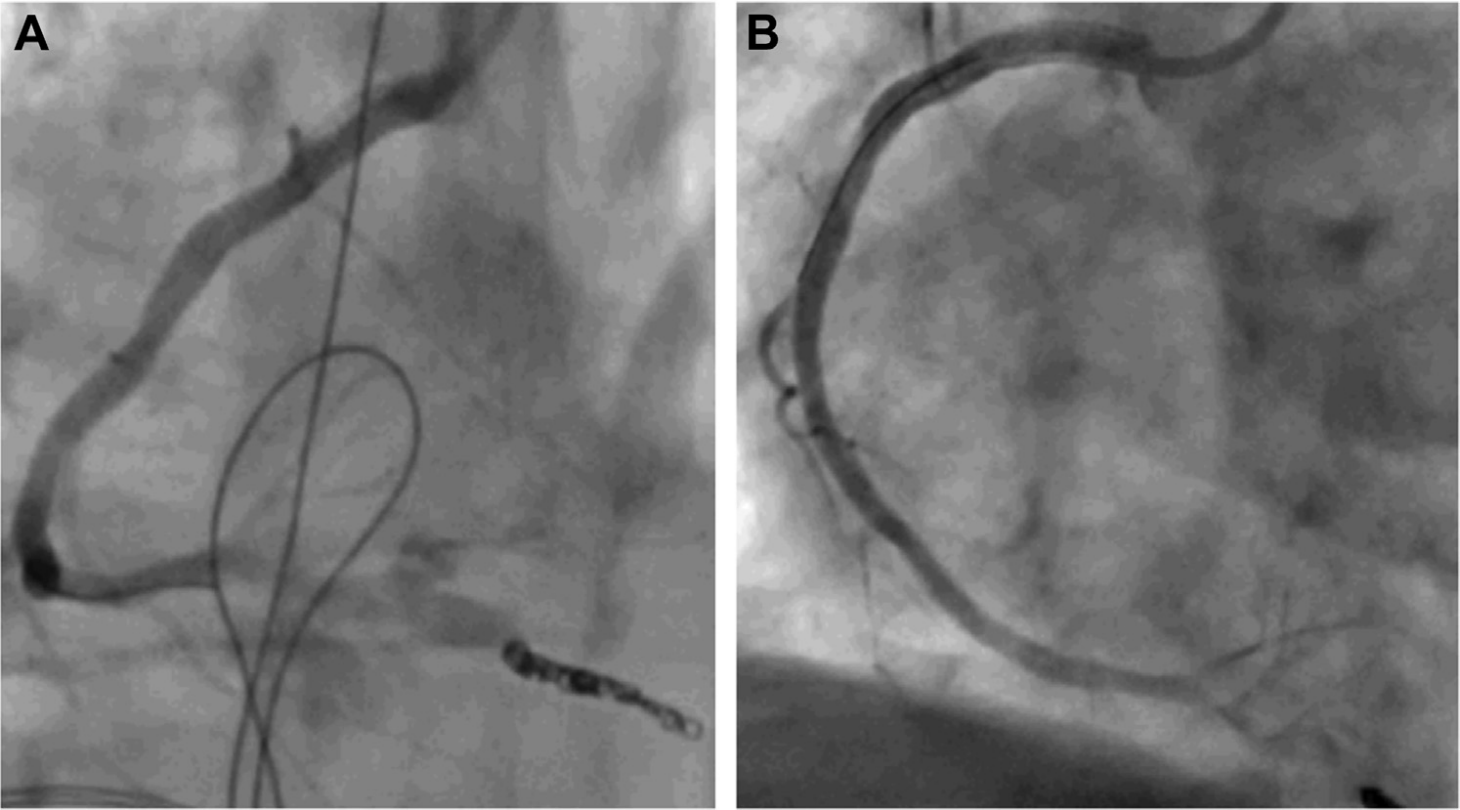
Final Angiogram (A) Final angiogram of the coiled posterior descending artery and the covered vessel rupture. (B) Final angiogram in left anterior oblique view with the reperfused proximal to distal right coronary artery.

This series of different hazards occurring within a single PCI procedure demanded several interventional bailout strategies. Distal perforation was resolved using coils and balloon occlusion, ischemia-triggered electrical storm was managed by immediate cardiopulmonary resuscitation, several defibrillations, and DES implantation to restore blood flow. Acute stent thrombosis was handled by correction of the activated clotting time, initially left at a suboptimal level due to distal perforation and ongoing bleeding. An attempt to cover a vascular rupture with a covered stent graft failed; however, spontaneous stabilization of the covered rupture occurred. Stent dislodgement was resolved using a self-made snaring device.^5^ Using a polymer-coated wire (Gladius Ex, ASAHI Intecc), it was possible to pass the entrapped stent graft in the proximal RCA and after dilatation with a 3.5-mm noncompliant balloon (Sapphire NC24, OrbusNeich), and a DES with high radial force (4.0 × 28-mm Synergy Megatron) was used to crush the stent graft to the vessel wall (Figures 7A and 7B). Rapid response of the resuscitation team helped to de-escalate the situation and allowed the intervention team to focus and resolve the case. Last, but not least, although the patient’s vital and myocardial capacities allowed for stabilization and termination of the case, prolonged myocardial ischemia (cumulative cardiopulmonary resuscitation duration >40 minutes), and suboptimal ventilation in the context of chronic obstructive cardiopulmonary disease GOLD stage IV, ultimately led to right heart failure. Escalation options such as right heart Impella device (Abiomed) were discussed but deemed not purposeful given the comorbidities and uncertain neurological outcome in respect of the patient decree. The patient eventually died as a consequence of an elective PCI where the 3 fundamentals of disaster met.

## Discussion

In general, frailty and chronic obstructive cardiopulmonary disease, as present in our patient, are predictors of poorer outcomes in patients undergoing PCI.^6^^,^^7^ In an all-comer, non-high-risk, non–chronic total occlusion population, the risk of coronary perforation is 0.39% (95% CI: 0.34%-0.45%) and is associated with a mortality of 7.5% (95% CI: 6.7%-8.4%).^8^ In Ellis type III perforations, in-hospital mortality is as high as 19%.^9^ Age alone seems not to be associated with an elevated risk for coronary perforation, but female sex (OR: 1.35; 95% CI: 1.30-1.41) and hypertension (OR: 1.21; 95% CI: 1.07-1.37) are.^8^ Furthermore, the use of a polymer-jacketed guidewire significantly increased the risk for distal vessel perforation.^10^ In our patient, 2 Ellis type III perforations occurred.^9^ Although state-of-the-art management with prolonged balloon occlusion followed by coiling were attempted, both interventions were unsuccessful and led to the vessel rupture with a larger balloon.^9^^,^^11^

This case illustrates how the snowball effect of a single “simple” complication can lead to a continuous chase of a series of complications. In hindsight, the following considerations might have hampered the risk and the impact of the hazard. First, using a microcatheter up front to escalate to a polymer-jacketed specialty wire and change back to a safe workhorse wire before any predilatation would have lowered the risk of distal perforation (hazard 1) and, in case of perforation, would have allowed rapid deployment of coils. Second, better lesion preparation of the proximal disease might have improved coronary flow and lowered the burden of prolonged coronary ischemia during the distal intervention (hazard 2), the risk of severe stent underexpansion with consecutive acute stent thrombosis (hazard 3), and stent graft entrapment (hazard 5). Third, activated clotting time should be kept in the therapeutic range irrespective of distal wire perforation (hazard 3). Fourth, for balloon occlusion, 1:1 sizing to the vessel diameter should be well respected to prevent vessel rupture (hazard 4).

## Conclusions

When patient-related vulnerability meets PCI-related complication hazards and interventional or patient-related capacities are insufficient, disastrous outcomes can and will occur.

**Visual Summary:**
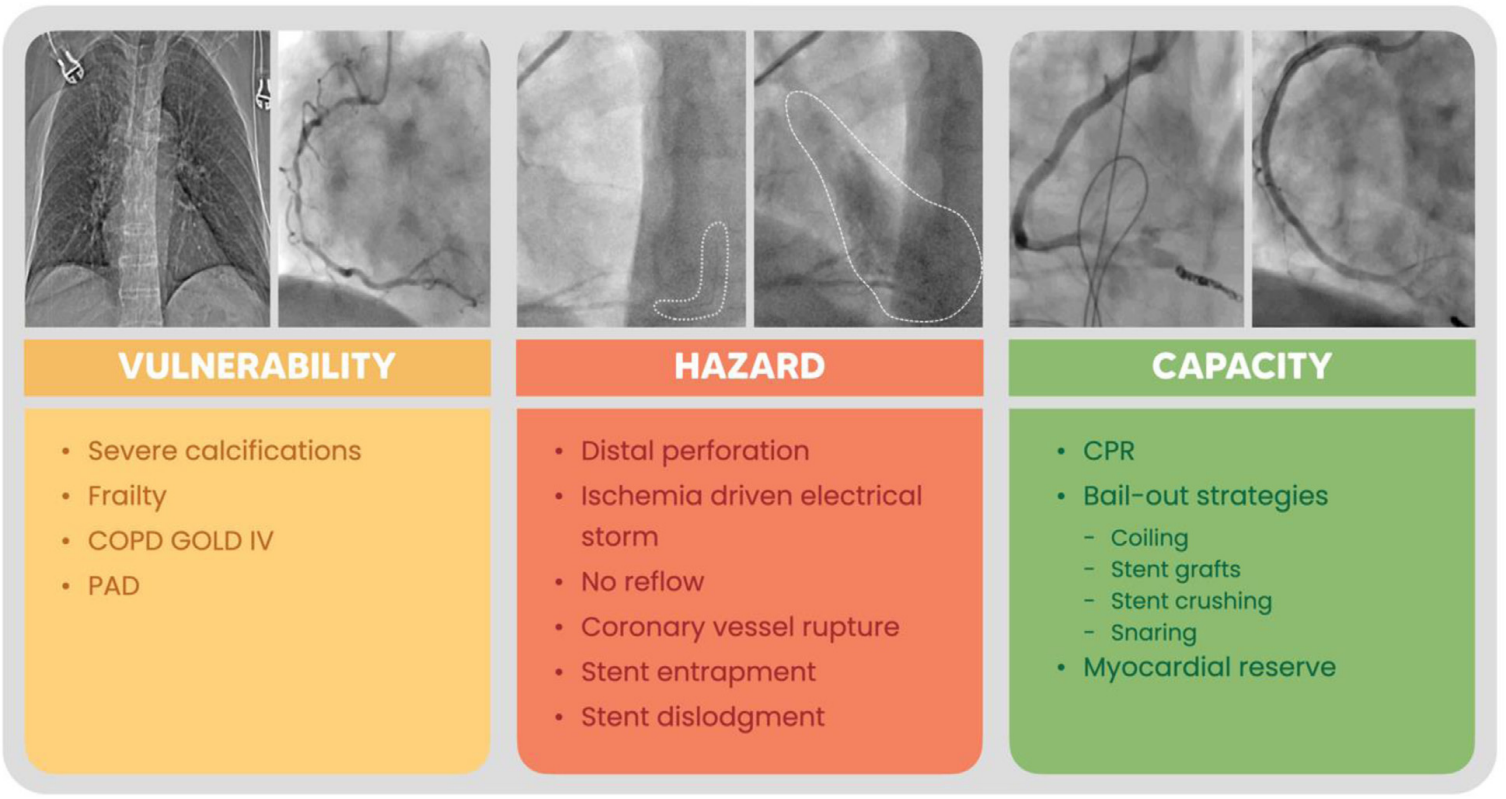
Disaster as a Function of Vulnerability, Hazard, and Capacity

## Funding Support and Author Disclosures

The authors have reported that they have no relationships relevant to the contents of this paper to disclose.
